# Supplementary material for: Enrichment of Tumor-Infiltrating B Cells in Group 4 Medulloblastoma in Children
Source: Int J Mol Sci. 2022 May 9;23(9):5287. doi: 10.3390/ijms23095287 (PMC9101625; doi:10.3390/ijms23095287)
Supplement: Supplementary file 1 [file ijms-23-05287-s001.zip › ijms-1628404-supplementary.pdf]

Table S1. Demography, molecular subgroup, clinical data, and molecular-clinical correlation in SickKids cohort of MBs.

**Molecular subgroup assignment**				
**N = 763**	**WNT n = 70 (18.9%)**	**SHH n = 223 (42.7%)**	**Group 3 n = 144 (29.2%)**	**Group 4 n = 326 (9.2%)**
**Age (median, range) at diagnosis (years)**				
8.0 (0.24–56.8)	10.8 (2–56.3)	8.8 (0.2–56.8)	5.1 (1.2–49.6)	8.0 (1.0–48.2)
≤3y (n = 119, 16.3%)	2 (3.1%)	70 (32.6%)	31 (22.8%)	16 (5.1%)
>3y (n = 610, 83.7%)	62 (96.9%)	145 (67.4%)	105 (77.2%)	298 (94.9%)
**Sex**				
Male, n = 472 (65.6%)	29 (45.3%)	128 (61.0%)	99 (72.3%)	216 (70.1%)
Female, n = 247 (34.4%)	35 (54.7%)	82 (39.0%)	38 (27.7%)	92 (29.9%)
Male/female ratio (1.9/1)	0.8/1	1.6/1	2.6/1	2.3/1
**Metastasis stage at diagnosis (M0-1, M2-3), number of cases (percentage)**				
M0, n = 397 (69.3%)	43 (87.8%)	134 (83.8%)	66 (60.6%)	154 (60.4%)
Met, n = 176 (30.7%)	6 (12.2%)	26 (16.2%)	43 (39.4%)	101 (39.6%)
**Pathology variant, number of cases (percentage) and median age (years)**				
Classic, n = 387 (66.0%), 8.0	40 (80.0%)	78 (43.1%)	68 (66.0%)	201 (79.8%)
DNMB, n = 109 (18.6%), 5.2	5 (10.0%)	73 (40.3%)	8 (7.8%)	23 (9.1%)
MBEN, n = 18 (3.1%), 2.4	0	10 (5.5%)	2 (1.9%)	6 (2.4%)
LCA, n = 72 (12.3%), 7.0	5 (10.0%)	20 (11.0%)	25 (24.3%)	22 (8.7%)
**Median follow-up time (range) (years)**				
3.9 (0–25.0)	4.2 (1.1–17.9)	3.9 (0.1–25.0)	3.0 (0–19.0).	4.2 (0–22.0)
**Survivals of molecular subgroup (percentage)**				
5-year OS rate: 74.9%	98.2%	79.1%	56.9%	75.0%

DNMB: Desmoplastic/nodular medulloblastoma, MBEN: Medulloblastoma with extensive nodularity, LCA: Large-cell/anaplastic, M0: no metastasis of tumors, Met: metastasis of tumors, OS: Overall survival.

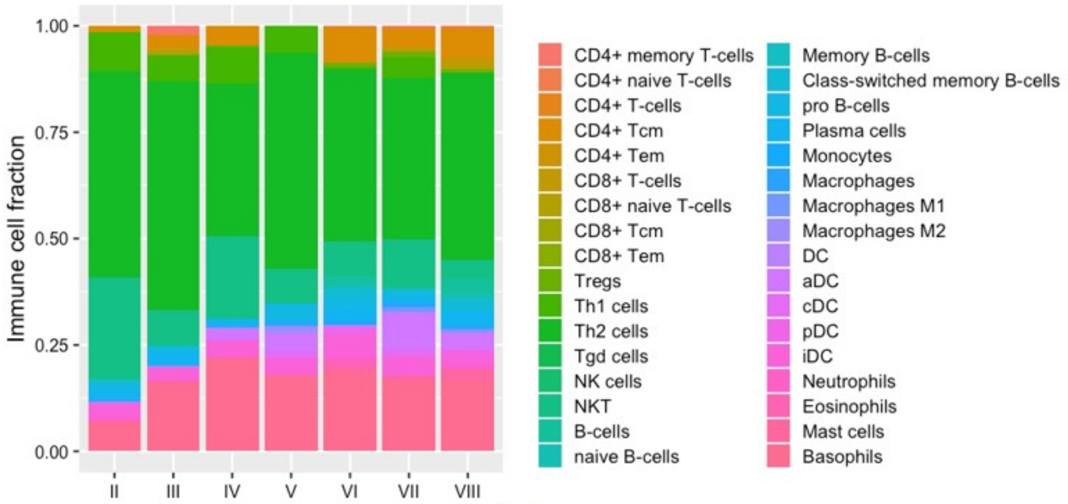

(a)

		WNT-α	WNT-β	SHH-α	SHH- β	SHH- γ	II	III	IV	V	VI	VII	VIII	MB	
Lymphoids	CD4+ memory T-cells	7.819e-03	0.000e+00	1.226e-03	2.750e-19	6.046e-19	1.533e-19	1.006e-02	6.805e-19	0.000e+00	4.894e-04	1.761e-03	1.978e-03	1.944e-03	-
	CD4+ naive T-cells	2.957e-04	7.978e-03	4.480e-04	5.189e-18	3.113e-18	4.419e-18	2.689e-03	1.047e-18	7.434e-18	1.004e-18	1.158e-18	1.949e-18	9.509e-04	-
	CD4+ T-cells	1.586e-18	0.000e+00	1.473e-18	2.300e-18	4.418e-19	1.222e-18	3.216e-20	4.169e-18	2.145e-19	1.191e-18	1.630e-18	4.259e-04	3.549e-05	-
	CD4+ Tcm	4.022e-02	4.408e-02	2.230e-03	1.399e-03	2.661e-03	8.214e-03	1.618e-02	2.581e-02	4.739e-18	4.249e-02	2.440e-02	3.209e-02	1.998e-02	- ***
	CD4+ Tem	7.941e-18	0.000e+00	4.643e-04	4.194e-03	2.326e-18	4.192e-18	2.772e-18	1.096e-17	3.233e-18	7.166e-18	3.907e-18	4.167e-18	3.882e-04	-
	CD8+ T-cells	1.220e-03	0.000e+00	9.707e-04	1.955e-18	4.038e-03	2.365e-03	7.985e-03	4.816e-19	5.123e-19	2.836e-19	3.920e-03	9.353e-03	2.488e-03	-
	CD8+ naive T-cells	3.073e-03	1.020e-18	2.998e-04	1.191e-03	4.611e-03	1.100e-18	2.205e-20	1.335e-18	5.712e-19	1.015e-18	9.728e-05	1.835e-03	9.256e-04	-
	CD8+ Tcm	4.958e-19	7.676e-19	1.404e-03	1.835e-18	8.439e-04	3.326e-18	2.027e-03	1.596e-18	1.178e-17	9.761e-19	9.550e-04	6.803e-19	4.358e-04	-
	CD8+ Tem	1.105e-19	0.000e+00	6.421e-19	3.606e-18	2.738e-18	1.342e-17	1.116e-18	1.201e-18	0.000e+00	1.336e-18	1.400e-18	1.622e-18	2.266e-18	-
	Tregs	5.797e-18	0.000e+00	2.996e-04	9.576e-03	1.018e-02	1.222e-18	3.396e-19	2.878e-03	2.531e-18	6.167e-03	6.525e-03	3.653e-03	3.273e-03	-
	Th1 cells	7.733e-03	1.842e-02	3.519e-02	1.121e-03	2.077e-02	6.402e-02	3.574e-02	4.900e-02	3.820e-02	2.126e-03	2.441e-02	2.411e-18	2.473e-02	- **
	Th2 cells	2.685e-01	1.725e-01	2.326e-01	1.386e-01	2.243e-01	3.366e-01	3.093e-01	2.060e-01	3.065e-01	2.026e-01	1.905e-01	1.999e-01	2.323e-01	-
	Tgd cells	6.639e-18	3.520e-18	3.033e-18	8.239e-18	5.328e-18	4.934e-18	0.000e+00	1.210e-17	0.000e+00	6.119e-18	3.028e-18	4.027e-18	4.747e-18	-
	NK cells	2.533e-18	1.656e-18	8.899e-18	5.085e-18	4.612e-18	1.155e-17	9.227e-19	0.000e+00	7.726e-18	3.930e-18	2.690e-18	1.313e-18	4.243e-18	-
	NKT	2.407e-03	7.460e-03	1.227e-02	1.150e-01	3.617e-02	1.668e-01	4.769e-02	1.121e-01	4.930e-02	4.057e-02	5.796e-02	1.997e-02	5.563e-02	- ***
	B-cells	0.000e+00	4.980e-04	3.134e-18	6.764e-19	1.608e-03	2.077e-03	3.888e-19	1.287e-18	0.000e+00	1.018e-02	1.532e-03	1.575e-02	2.637e-03	- *
	naive B-cells	3.805e-04	8.021e-03	8.556e-19	7.582e-18	5.500e-19	1.759e-18	8.164e-04	3.401e-19	0.000e+00	4.499e-03	9.342e-04	5.045e-03	1.641e-03	- *
	Memory B-cells	5.847e-18	2.370e-19	9.305e-18	1.443e-17	3.903e-18	1.022e-03	7.765e-18	5.332e-18	0.000e+00	1.646e-03	1.339e-04	9.295e-04	3.109e-04	-
	Class-switched memory B-cells	4.022e-19	0.000e+00	3.928e-18	8.513e-18	1.187e-18	4.270e-03	1.135e-03	2.924e-18	8.002e-19	1.114e-02	3.511e-03	1.195e-02	2.667e-03	- ***
	pro B-cells	4.774e-18	3.113e-19	1.608e-03	4.027e-20	3.273e-03	1.217e-02	7.367e-03	2.351e-19	2.496e-02	1.180e-02	4.631e-03	2.165e-03	5.664e-03	- **
	Plasma cells	5.354e-03	6.131e-03	2.627e-03	2.529e-03	4.619e-03	1.788e-02	1.710e-02	1.282e-02	1.753e-03	1.476e-02	8.749e-03	1.508e-02	9.117e-03	- ***
Myeloids	Monocytes	1.123e-02	7.951e-18	4.005e-03	1.208e-02	1.743e-18	1.720e-19	3.494e-19	1.358e-17	0.000e+00	4.049e-03	2.936e-18	3.990e-18	2.613e-03	-
	Macrophages	1.915e-02	1.097e-02	1.328e-02	5.357e-02	1.878e-03	4.076e-19	3.910e-19	3.668e-18	3.237e-03	4.613e-19	3.216e-03	2.178e-03	8.956e-03	- *
	Macrophages M1	1.474e-02	1.442e-02	2.118e-02	5.298e-02	9.292e-03	3.181e-19	3.240e-04	6.890e-06	2.727e-03	1.470e-04	5.375e-03	1.775e-03	1.025e-02	- ***
	Macrophages M2	1.523e-02	0.000e+00	6.498e-03	2.177e-02	1.599e-03	2.241e-19	1.265e-03	2.873e-19	8.556e-03	4.520e-20	1.194e-03	2.669e-03	4.898e-03	- **
	DC	2.815e-03	8.044e-04	1.033e-03	5.755e-03	9.060e-04	6.920e-20	1.941e-19	8.267e-19	2.798e-19	5.518e-19	6.909e-04	1.166e-19	1.000e-03	-
	aDC	5.541e-02	4.017e-02	1.041e-01	7.822e-02	6.679e-02	1.068e-18	6.416e-19	1.551e-02	2.701e-02	2.140e-03	4.358e-02	1.585e-02	3.739e-02	- *
	cDC	4.717e-02	4.344e-02	3.285e-02	7.540e-02	2.043e-02	3.887e-03	1.846e-03	7.599e-04	8.326e-03	2.709e-03	7.162e-03	2.456e-03	2.054e-02	- ****
	pDC	9.102e-19	1.329e-03	4.045e-03	1.624e-03	4.683e-18	5.020e-19	4.153e-04	3.246e-03	3.668e-04	6.995e-03	1.851e-03	4.288e-03	2.013e-03	- **
	iDC	4.717e-02	1.162e-01	2.453e-02	1.345e-01	1.930e-02	2.608e-02	1.500e-02	1.933e-02	2.310e-02	2.457e-02	2.168e-02	1.323e-02	4.039e-02	-
	Neutrophils	6.434e-03	0.000e+00	1.762e-19	4.788e-03	4.407e-04	2.817e-04	2.791e-04	1.013e-03	5.678e-20	8.189e-03	3.478e-04	1.717e-03	1.958e-03	-
	Eosinophils	1.411e-18	0.000e+00	3.884e-19	2.672e-03	4.661e-19	3.625e-18	2.298e-03	4.335e-19	3.749e-18	5.701e-03	8.965e-04	2.434e-03	1.167e-03	-
	Mast cells	5.385e-03	7.085e-03	4.845e-03	8.796e-04	3.212e-03	5.557e-03	5.928e-03	3.977e-03	1.026e-03	6.471e-03	5.475e-03	1.081e-02	5.054e-03	- *
	Basophils	1.519e-02	1.303e-01	3.034e-02	1.299e-02	1.977e-02	4.498e-02	8.805e-02	1.230e-01	1.079e-01	9.211e-02	8.289e-02	7.472e-02	6.852e-02	- ***

(b)

Figure S1. Analysis of immune cells and B cell subsets from the 70 MB patients based on the 2021 WHO classification of MB subgrouping. (**a**) The proportion of T cells, B cells, macrophages, DCs, mast cells and other myeloid cells in MB tumors. (**b**) The distribution of lymphoid and myeloid cells enrichment.

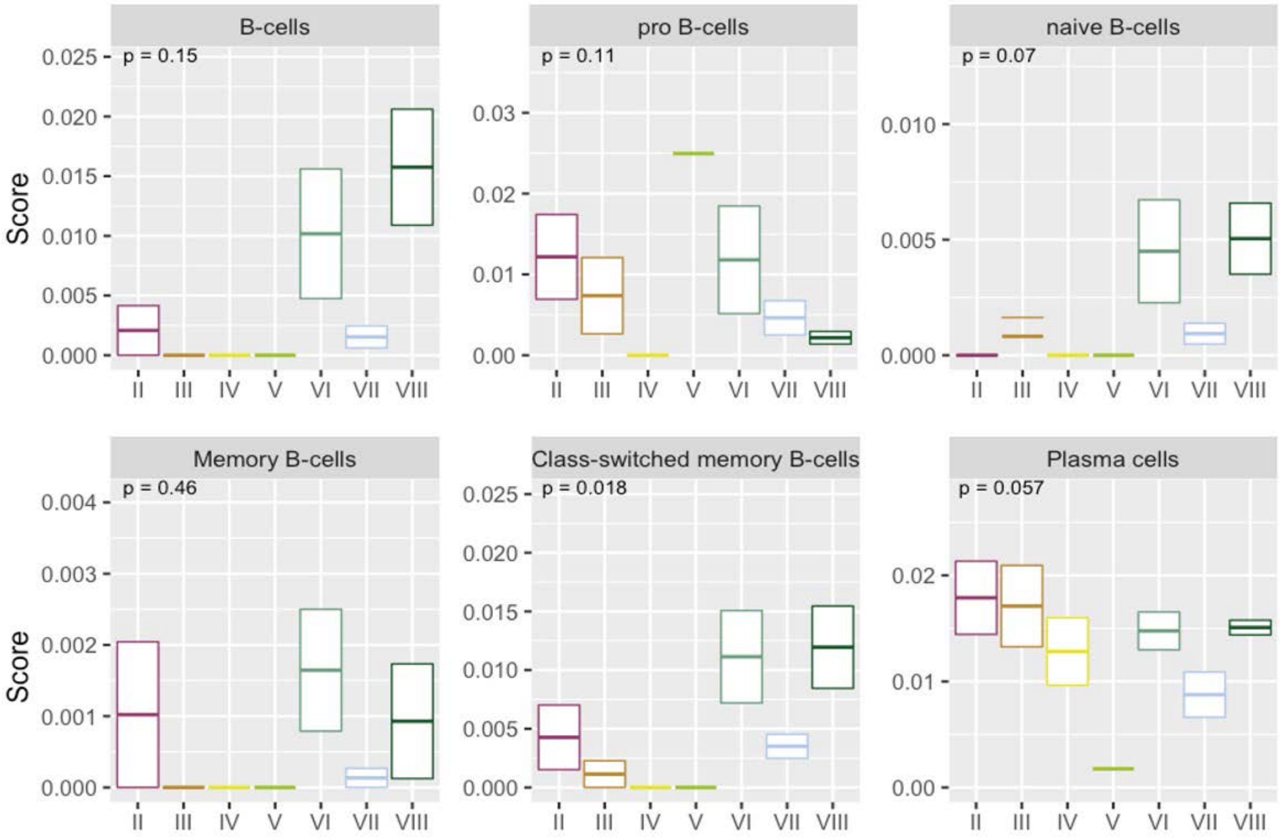

Figure S2. Expression of the infiltrating B cells subsets from in the MB subgroups. The analysis of B cell subsets from the 70 clinical patients based on the 2021 WHO classification of MB. The p values were determined by Kruskal-Wallis test.

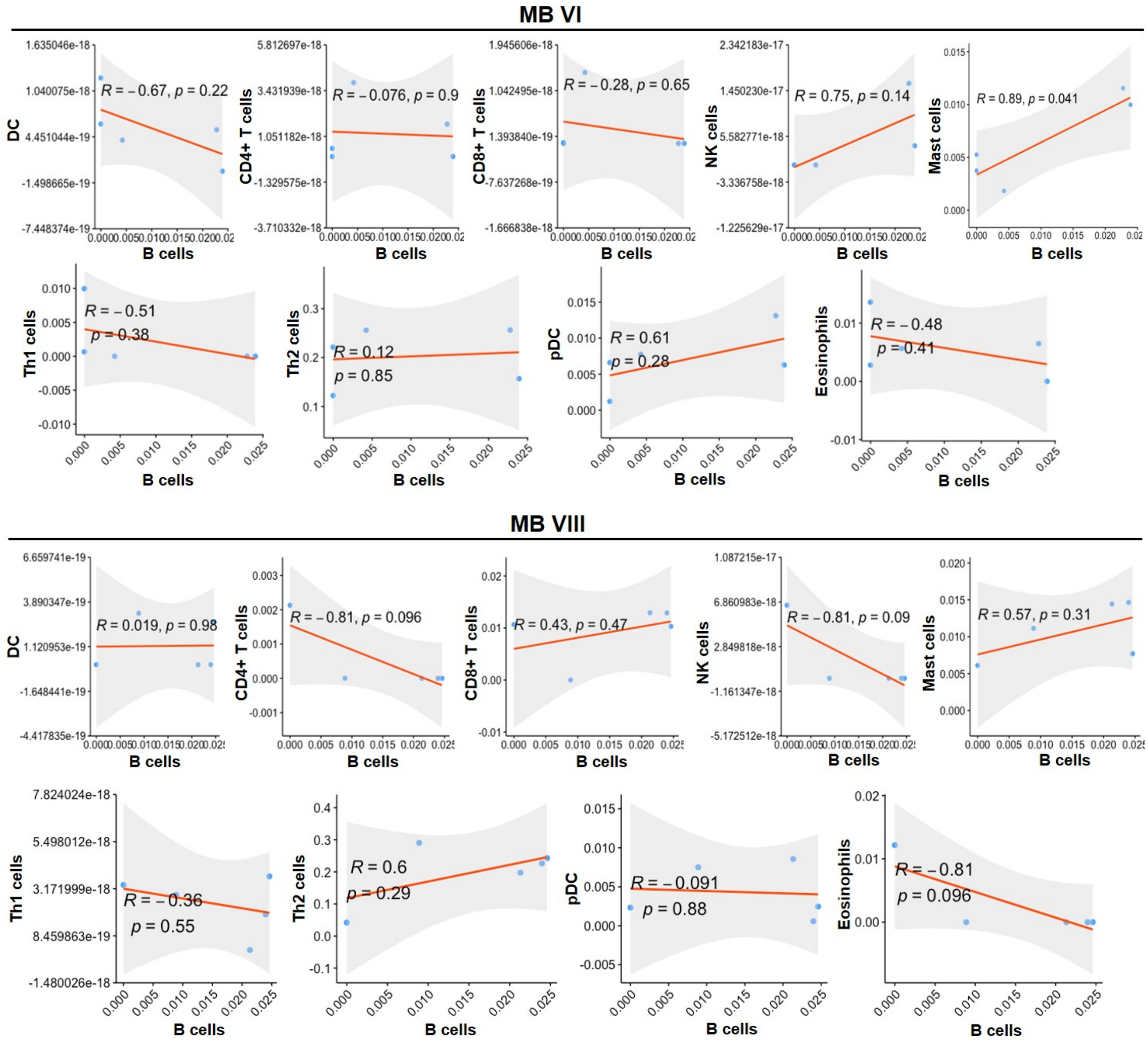

Figure S3. Correlation of B cells with other immune cell types in the VI and VIII subgroups of MB tumors. Correlation between the expression of B cells and the expression of DCs, $CD4^+$ T cells, $CD8^+$ T cells, NK cells, mast cells, Th1 cells, Th2 cells, pDCs and eosinophils in the VI and VIII subgroups of MB tumors. Correlation coefficients (R) were obtained by using Pearson's correlation coefficient test.

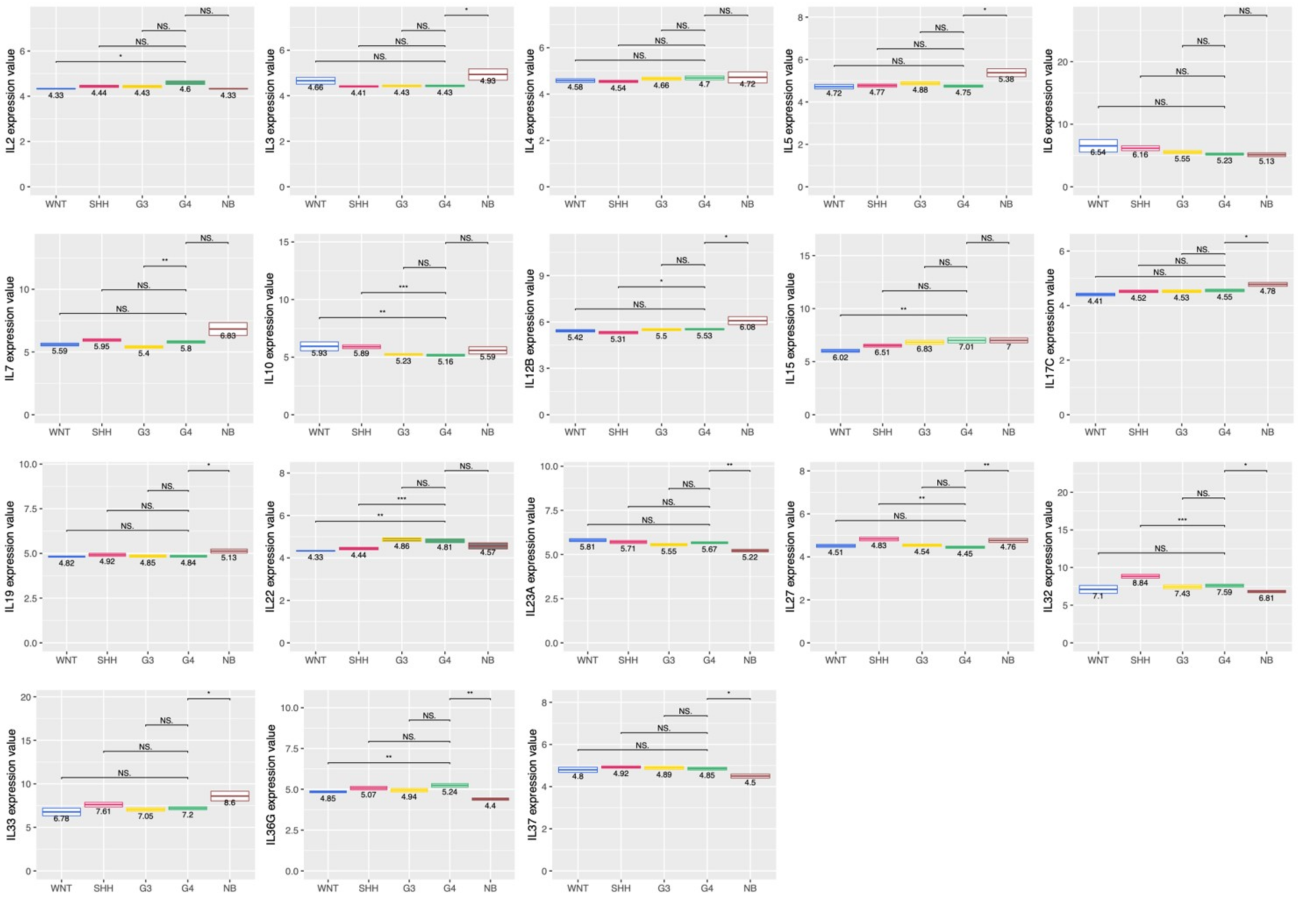

Figure S4. Expression of interleukins in different MB subgroups tumors and normal brain. The expressions of interleukins among 4 subgroups of MB tumors and normal brain tissue were analyzed from the transcriptome data. *, $p < 0.05$; **, $p < 0.01$; ***, $p < 0.001$, compared to G4 subgroup tumors. NS: no significant difference.

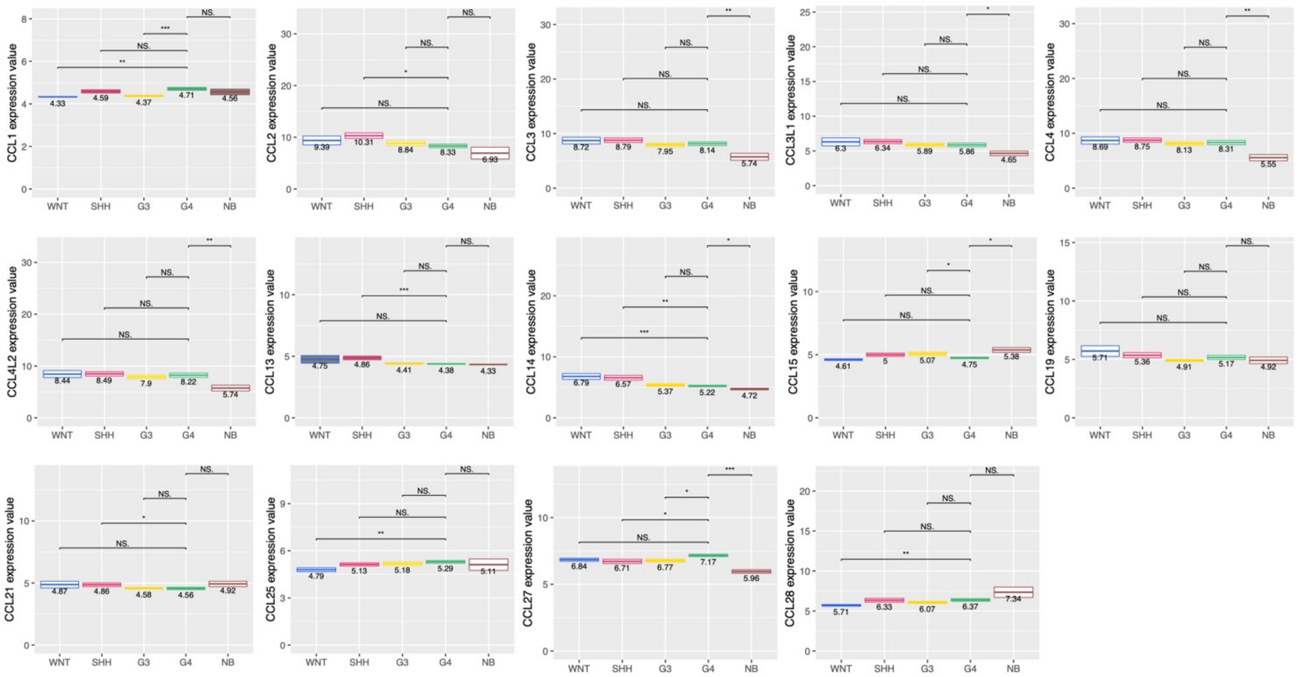

Figure S5. Expression of CCL chemokines in different MB subgroups tumors and normal brain. The expressions of CCL chemokines among 4 subgroups of MB tumors and normal brain tissue were analyzed from the transcriptome data. *, $p < 0.05$; **, $p < 0.01$; ***, $p < 0.001$, compared to G4 subgroup tumors. NS: no significant difference.

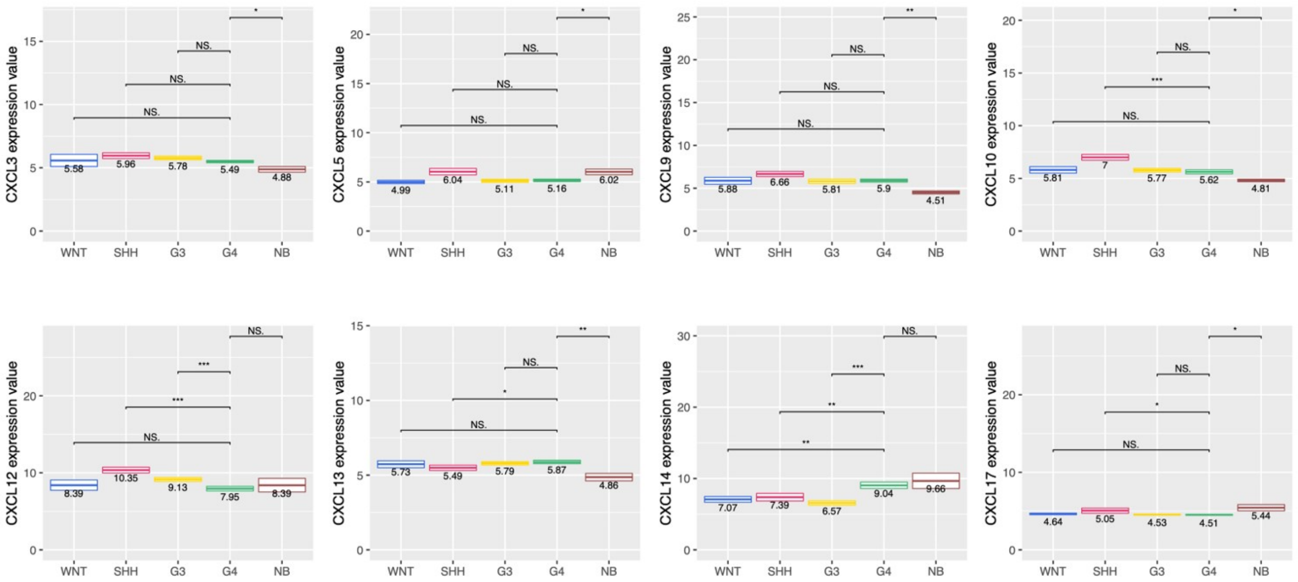

Figure S6. Expression of CXCL chemokines in different MB subgroups tumors and normal brain. The expressions of CXCL chemokines among 4 subgroups of MB tumors and normal brain tissue were analyzed from the transcriptome data. *, $p < 0.05$; **, $p < 0.01$; ***, $p < 0.001$, compared to G4 subgroup tumors. NS: no significant difference.
